# Editorial

**DOI:** 10.1590/S1678-77572010000100001

**Published:** 2010

**Authors:** Carlos F. Santos

**Affiliations:** Associate Professor. Editor-in-Chief. Journal of Applied Oral Science

Dear Readers, Contributors and Reviewers,

I would like to take this opportunity to wish all of you an excellent year in 2010. It is also important to thank all of you for your kindness, support, patience and interest in the Journal of Applied Oral Science (JAOS) in 2009.

After the indexation in the two most prestigious databases (PubMed/MEDLINE and ISI Web of Knowledge), the JAOS was flooded with a huge number of submissions. My small team and I were quite happy and honored with such a demonstration of confidence in our work, but we were not ready to handle that enormous number of submissions. Therefore, I decided to temporarily block our online system for new submissions in order to properly process and analyze all the submissions. I hope all the contributors understand that this tough decision was actually a demonstration of respect to their works. Soon the system will be reopened and some changes will be added. The main new requirement will be that in the initial submission, all the contributors will be asked to provide the JAOS with the names and e-mail addresses of four potential reviewers since this is, in my opinion, the bottleneck of the reviewing process. This should help to speed up the reviewing process.

This year the JAOS will have its first impact factor (IF) from the Journal Citation Report (JCR). I am aware we will have a low IF, but this will be instrumental to guide and encourage us to improve it. I count on everyone’s help, support and suggestions to face this challenge!

Finally, I thank all the reviewers and our editorial board, who kindly and blindly evaluated works for possible publication in the JAOS in 2009. This silent and generous work is the most important and delicate in the reviewing process, and without this precious help our journal would not have reached the international respect it currently has.

Please, feel free to contact me through the new e-mail address: editorjaos@fob.usp.br. We also invite all readers, contributors and reviewers to visit our free complete online collection and our online submission system at http://www.scielo.br/jaos.

Kind regards,

